# The chemistry of amine radical cations produced by visible light photoredox catalysis

**DOI:** 10.3762/bjoc.9.234

**Published:** 2013-10-01

**Authors:** Jie Hu, Jiang Wang, Theresa H Nguyen, Nan Zheng

**Affiliations:** 1 Department of Chemistry, University of Arkansas, Fayetteville, AR 72701, USA

**Keywords:** α-amino radical, amine radical cation, catalysis, distonic ion, free radical, iminium ion, photoredox, visible light

## Abstract

Amine radical cations are highly useful reactive intermediates in amine synthesis. They have displayed several modes of reactivity leading to some highly sought-after synthetic intermediates including iminium ions, α-amino radicals, and distonic ions. One appealing method to access amine radical cations is through one-electron oxidation of the corresponding amines under visible light photoredox conditions. This approach and subsequent chemistries are emerging as a powerful tool in amine synthesis. This article reviews synthetic applications of amine radical cations produced by visible light photocatalysis.

## Introduction

Amine radical cations, which are an odd-electron species, are of great utility in amine syntheses [1–8]. They can be formed by loss of an electron from the corresponding amines. This one-electron oxidation process has been realized by using electrochemistry [9–11], chemical oxidants [12–14], metal-catalyzed oxidation [15–18], UV light-mediated photochemistry [7,19–21], and visible light-mediated photochemistry [22–23]. Recently, the last approach has become a major research focus in organic chemistry. The enthusiasm surrounding this approach is partially driven not only by its green characteristics (i.e. using visible light), but also more importantly by its unique ability to achieve unconventional bond formation.

Like most organic compounds, amines do not absorb visible light efficiently, unless they have a chromophore (e.g., conjugated π-bond systems). Therefore, a photocatalyst is often required to initialize electron-transfer reactions with amines. Some of the frequently used photocatalysts include ruthenium [24–26] and iridium [27–28] polypyridyl complexes as well as organic dyes [29–30] that are absorbed in the visible-light region. They all share one common characteristic: a facile intersystem crossing (ISC) that allows the conversion of the initially formed singlet photoexcited state to the relatively long-lived triplet photoexcited state. The triplet photoexcited state’s long lifetime permits it to engage in single-electron transfer with organic molecules such as amines. The photoexcited state is both more oxidizing and more reducing than the ground state. It can be quenched reductively by accepting an electron from an electron donor or oxidatively by donating an electron to an electron acceptor. Amines are often used as an electron donor to reductively quench the photoexcited state while they are oxidized to amine radical cations. This single-electron transfer process was investigated intensively in the late 1970s and early 1980s because amines were used as a sacrificial electron donor in water splitting [31–32] and carbon dioxide reduction [33–34]. Since 2008, seminal works from MacMillan, Yoon, and Stephenson have reinvigorated the field of visible light photoredox catalysis [35–42]. The use of amines as both the electron donor and the substrate, rather than just the electron donor, has become a major approach to exploit synthetic utility of photogenically produced amine radical cations.

**Scheme 1:**
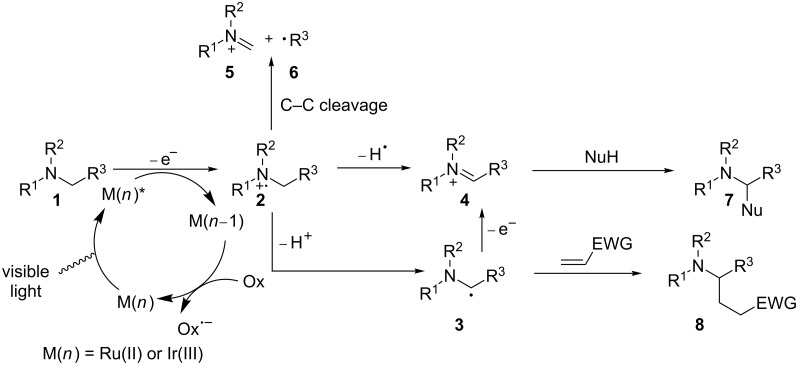
Amine radical cations’ mode of reactivity.

Reductive quenching of the photoexcited state of a photocatalyst (M) by amine **1** is governed by the reduction potentials of the photoexcited state and the amine (Scheme 1). The amine’s reduction potential, which can be readily measured by cyclic voltammetry, should be less positive than that of the photoexcited M. The solvent also has a significant impact on the oxidation and the subsequent reactions [43–44]. A polar solvent is generally favored for electron-transfer reactions involving amine radical cations, but identification of the optimal solvent requires experimentation. Once formed, amine radical cation **2** has been shown to have four modes of reactivity. The first mode is the back electron transfer reaction, which involves amine radical cation **2** giving back one electron to M(*n*−1). This is a major side reaction competing against the other productive downstream reactions of **2**. To circumvent this side reaction, two approaches or a combination thereof can be exploited [45–46]. One approach involves modifying the structure of the ligand on M to retard the back electron transfer. The other involves designing fast and/or irreversible downstream reactions of **2**. The second mode involves hydrogen atom abstraction from **2** to produce iminium ion **4**, when a good hydrogen atom acceptor is present in the reaction. The use of amine radical cation **2** as the source of a hydrogen radical has been applied to a number of visible light-mediated reductions such as reductive dehalogenation [47–51], reductive radical cyclization [52–54], reduction of activated ketones [49], and reduction of aromatic azides [55]. The third mode involves deprotonation of amine radical cation **2** to form α-amino radical **3**, which is converted to iminium ion **4** by another one-electron oxidation. The acidifying effect of one-electron oxidation on the α-C–H bond remains debatable [56–60]. The rate for deprotonation of amine radical cation **2** has been measured experimentally by several groups, and a broad range has been obtained [61–62]. α-Amino radical **3** is strongly reducing [45,63], thus making the second one-electron oxidation facile. The last mode involves cleavage of a C–C bond α to the nitrogen atom, yielding a neutral free radical **6** and iminium ion **5**. Iminium ion **4**, an excellent electrophile, is amenable to interception by a variety of nucleophiles to directly install a new bond at the position α to the nitrogen atom. In contrast, α-amino radical **3** is nucleophilic. It tends to add to electron-deficient alkenes to form a C–C bond, also at the position α to the nitrogen atom.

This review will summarize the work to date on the use of amine radical cations generated under visible light photoredox conditions as a key intermediate to trigger downstream reactions. The work is grouped based on the key intermediates (iminium ions and α-amino radicals) or processes (cleavage of C–C and N–N bonds) involved. The chemistries that have focused on the use of amines as a sacrificial electron donor only or as a hydrogen radical donor only will not be discussed in the review. These chemistries have been recently reviewed [22–23,35–42]. Photooxidation of amines to amine radical cations can also be achieved using UV light with a sensitizer. This approach and subsequent chemistries are also outside the scope of this review. Interested readers are referred to these reviews [7,19–21].

## Review

### Iminium ions

#### Intercepted by carbon nucleophiles

One of the major modes of reactivity for amine radical cations is their conversion to the powerful electrophilic iminium ions, which can be intercepted by a range of pronucleophiles to form a number of important bonds such as C–C, C–N, C–O, and C–P. The chemistry involving iminium ions has seen the most synthetic applications so far.

**Scheme 2:**
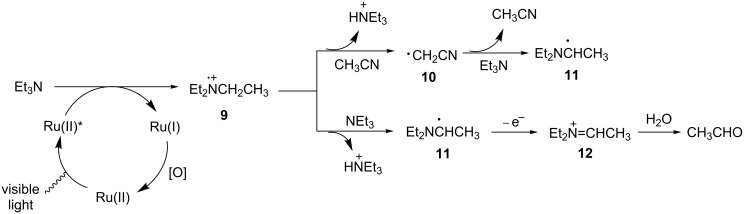
Reductive quenching of photoexcited Ru complexes by Et_3_N.

The Whitten group provided some early studies to establish the conversion of amine radical cations to iminium ions. In 1980, Giannotti and Whitten reported that irradiation of triethylamine with three ruthenium polypyridyl complexes using visible light in the presence of water yielded acetaldehyde, presumably formed by the hydrolysis of iminium ion **12** (Scheme 2) [46]. They proposed that reductive quenching of the photoexcited Ru(II) complex by triethylamine produced Ru(I) and amine radical cation **9**. Then amine radical cation **9** can either abstract a hydrogen atom from the solvent (CH_3_CN) to form carbon radical **10**, or lose a proton to another molecule of triethylamine to form α-amino radical **11**. Carbon radical **10** is converted to α-amino radical **11** by abstracting a hydrogen atom from a second molecule of triethylamine and CH_3_CN is ultimately regenerated. Finally, one electron oxidation of α-amino radical **11** furnishes iminium ion **12** that is hydrolyzed to acetaldehyde. Although the authors were not able to detect amine radical cation **12** spectroscopically, they were able to use ESR (electron spin resonance) techniques to detect Ru(I) and α-amino radical **11** with the aid of a spin trap, nitrosodurene.

**Scheme 3:**
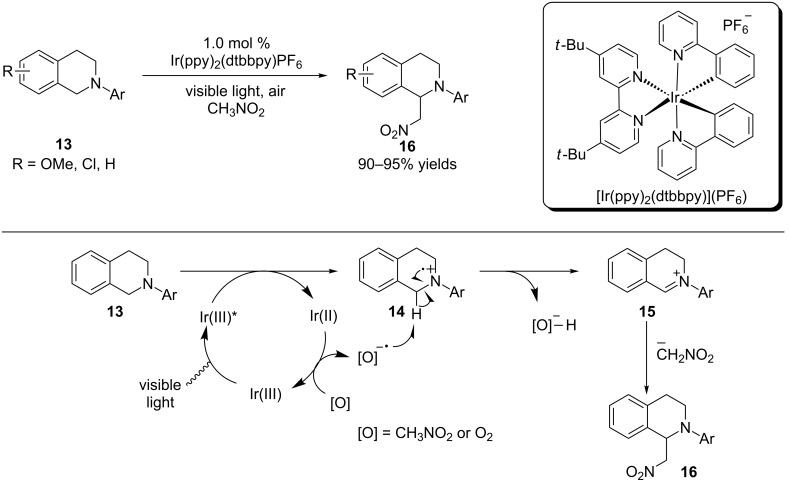
Photoredox aza-Henry reaction.

In 2010, Stephenson and coworkers reported a visible light-mediated aza-Henry reaction that harnesses the synthetic potential of iminium ions. Using only 1 mol % of [Ir(ppy)_2_(dtbbpy)](PF_6_) and visible light, a variety of *N*-aryltetrahydroisoquinolines were oxidatively coupled with nitroalkanes to provide the aza-Henry products in excellent yields (Scheme 3) [64]. They suggested that reductive quenching of the Ir(III) photoexcited state by *N*-aryltetrahydroisoquinolines **13** leads to the formation of amine radical cation **14** and the powerful reducing agent Ir(II) (Ir(III)/Ir(II), −1.51 V vs SCE). The Ir(II) catalyst then reduces nitromethane or oxygen to a radical anion that may abstract a hydrogen atom from amine radical cation **14** to form the iminium ion **15**. Interception of the iminium ion by the anion of nitromethane affords the aza-Henry product **16**.

**Scheme 4:**
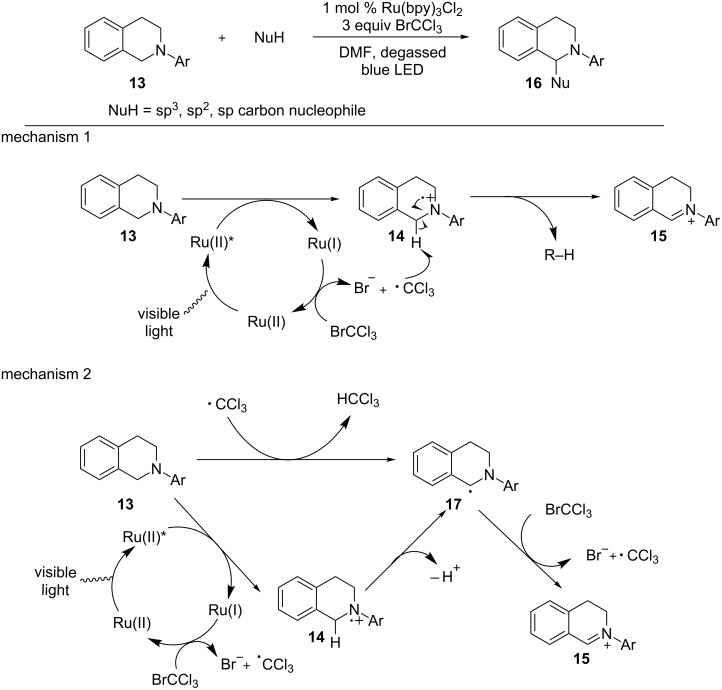
Formation of iminium ions using BrCCl_3_ as stoichiometric oxidant.

Oxygen has been the most often used stoichiometric oxidant in the formation of iminium ions under photoredox conditions. However, this use has some limitations. The catalyst turnover mediated by O_2_ is often slow, resulting in long reaction time. O_2_ can also intercept α-amino radicals, one of the key intermediates in the formation of iminium ions, to produce amides and thus compromise the formation of iminium ions [65–66]. The Stephenson group sought an alternative stoichiometric oxidant to overcome the limitations encountered by O_2_ (Scheme 4) [58]. BrCCl_3_ was identified as such an alternative and iminium ions were produced cleanly within 3 hours. A broad range of nucleophiles, including nitroalkanes, was shown to add to iminium ions. The authors proposed two possible mechanisms for the formation of iminium ions based on the two divergent pathways for the conversion of amine radical cations to iminium ions. The first mechanism is based on the pathway involving abstraction of a hydrogen atom from amine radical cation **14**. The hydrogen atom acceptor is a trichloromethyl radical, which is formed via one-electron reduction of BrCCl_3_ by Ru(I). The second is centered on the pathway involving deprotonation of amine radical cation **14** followed by one-electron oxidation. BrCCl_3_ is the one-electron oxidant via electron transfer or atom transfer. The trichloromethyl radical, which is generated by this oxidation, then abstracts a hydrogen atom of another molecule of *N*-aryltetrahydroisoquinoline to produce the α-amino radical **17**, which once again enters the radical chain process with BrCCl_3_.

**Scheme 5:**
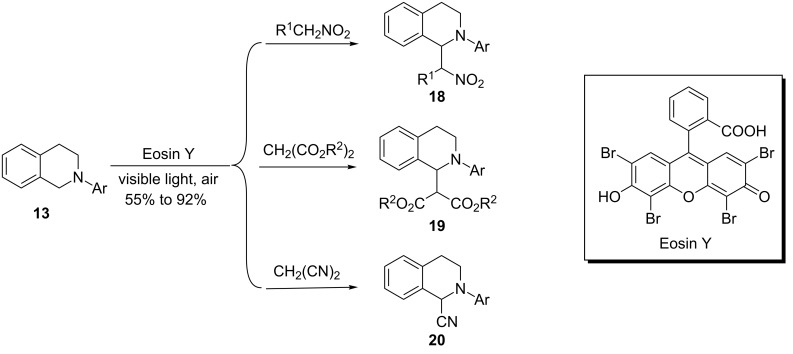
Oxidative functionalization of *N*-aryltetrahydroisoquinolines using Eosin Y.

König and coworkers showed that the same aza-Henry reaction can be catalyzed by the organic dye Eosin Y to afford the aza-Henry product **18** (Scheme 5) [67]. In addition to nitroalkanes, dialkyl malonates and malononitrile can be used as pronucleophiles to provide β-diester amine **19** and α-aminonitrile **20**. The authors proposed a mechanism similar to that proposed by Stephenson and coworkers for the aza-Henry reaction catalyzed by the Ir complex (Scheme 3). The Tan group simultaneously reported that another organic dye, Rose Bengal (RB), can be used in place of Eosin Y to catalyze the aza-Henry reaction [68].

**Scheme 6:**
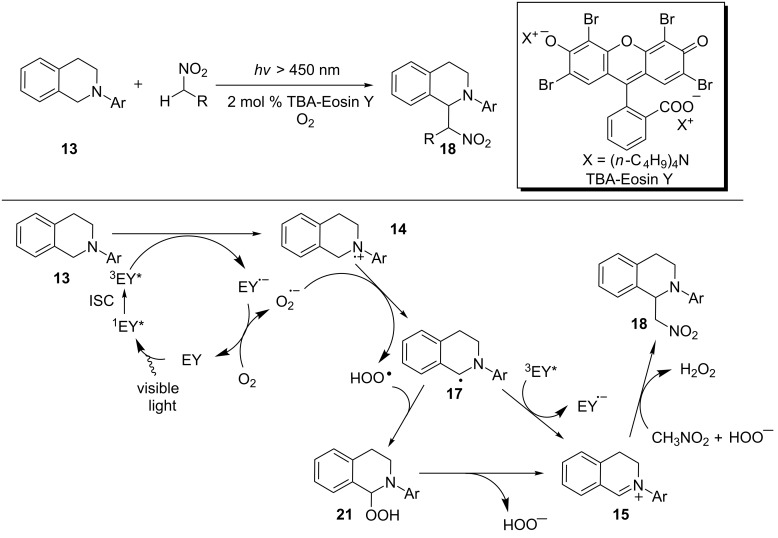
Synthetic and mechanistic studies of Eosin Y-catalyzed aza-Henry reaction.

The Wu group concurrently developed the Eosin Y-catalyzed aza-Henry reaction as reported by König and also performed mechanistic studies on the reaction. Their proposed catalytic cycle for the reaction is detailed in Scheme 6 [69]. Wu and coworkers were able to obtain experimental evidence to lend support to some of the key steps in the catalytic cycle. An oxygen uptake experiment showed that 0.75 equiv of O_2_ was consumed for the complete conversion of *N*-phenyltetrahydroisoquinoline **13**. This data strongly supports the role of O_2_ as the stoichiometric oxidant. Flash photolysis studies established that reductive quenching of the triplet excited state of Eosin Y by *N*-phenyltetrahydroisoquinoline **13** produced the Eosin Y radical anion. An ESR study on the irradiated solution of DMPO (5,5-dimethyl-1-pyrroline-*N*-oxide), Eosin Y, and *N*-phenyltetrahydroisoquinoline in air-saturated CH_3_CN detected the adduct of superoxide to DMPO. In contrast, an ESR study on the same solution but with DMPO being replaced by TEMP (2,3,6,6-tetramethylpiperidine) did not detect TEMPO, the oxidation product of TEMP by singlet oxygen. However, TEMPO was detected in the absence of *N*-phenyltetrahydroisoquinoline. The results from these ESR studies are consistent with the notion that singlet oxygen is not formed in the presence of *N*-phenyltetrahydroisoquinoline and the Eosin Y radical anion reduces oxygen to superoxide. Finally, the yield of the product **18** increased when the reaction mixture was kept stirring in the dark after 4 h irradiation. This observation supports the formation of hydroperoxide intermediate **21**.

**Scheme 7:**
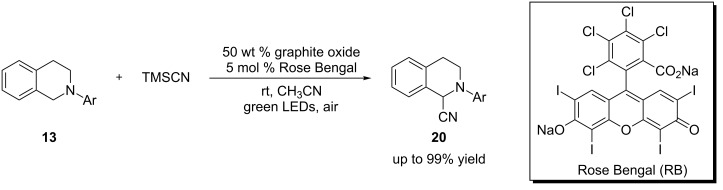
Oxidative functionalization of *N*-aryltetrahydroisoquinolines using RB and GO.

Tan and coworkers employed a cocatalyst system composed of the organic dye Rose Bengal and graphite oxide (GO) for α-cyanation of *N*-aryltetrahydroisoquinolines (Scheme 7) [70]. The use of GO as carbocatalyst, pioneered by the Bielawski group, has been shown to facilitate a variety of reactions including oxidation, reduction, dehydration, and C–C bond formation [71–74]. GO was found to improve the yields of the α-cyanation reaction, and this was the first example of using GO to promote visible light-mediated reactions. The synergistic effect between carbocatalysis and visible light-mediated photocatalysis has the potential to be further explored in other photocatalyzed reactions.

Since visible light photocatalysis is often orthogonal to or compatible with a number of common catalytic processes, merging it with another type of catalysis has become a recent development in the field of visible light photocatalysis. One direct benefit of this dual catalysis approach is to allow expansion of the types of nucleophiles capable of adding to the iminium ions generated under photoredox conditions.

**Scheme 8:**
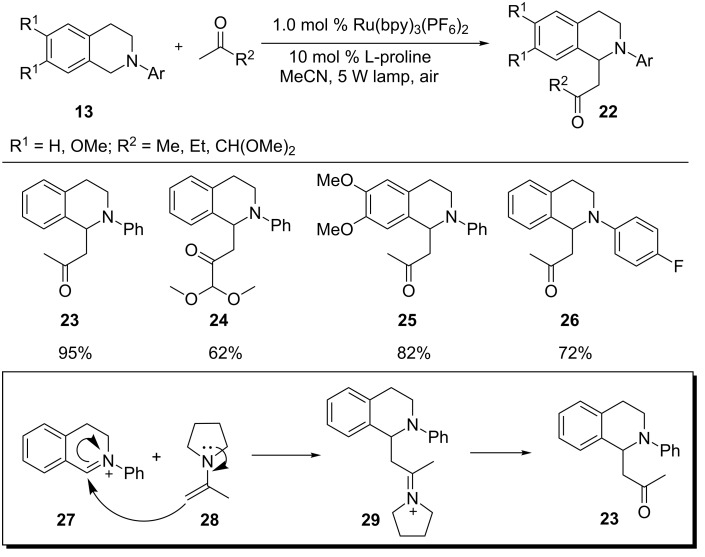
Merging Ru-based photoredox catalysis and Lewis base catalysis for the Mannich reaction.

In 2011, Rueping and coworkers described a dual catalytic system combining photoredox and Lewis base catalysis for the functionalization of C–H bonds α to the nitrogen atom of *N*-aryltetrahydroisoquinoline **13** (Scheme 8) [65]. In the presence of a Lewis base, a ketone is converted to enamine nucleophile **28** in situ, which is then added to photogenically formed iminium ion **27** to yield the Mannich product **23**. The Mannich reaction was sluggish without the Lewis base, and a side reaction, formation of the oxidized isoquinoline, became significant. The choice of Lewis base was found to be also crucial for the outcome of the reaction and proline was more effective than pyrrolidine. Additionally, to maximize the yields, the optimal rates for the two catalytic processes need to be similar. Since formation of the iminium ions is much faster than the addition of the enamine nucleophiles, higher yields were realized with slower formation of the iminium ions. This was achieved by use of [Ru(bpy)_3_](PF_6_)_2_ in conjunction with a weak light source (5 W fluorescence bulb).

**Scheme 9:**
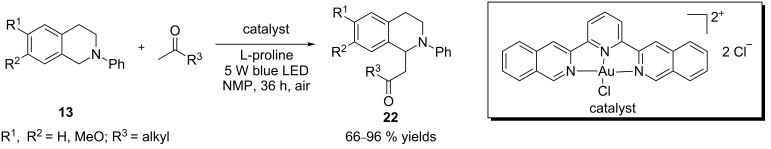
Merging Au-based photoredox catalysis and Lewis base catalysis for the Mannich reaction.

The Che group synthesized a photoactive gold(III) complex that was shown to catalyze α-cyanation of *N*-aryltetrahydroisoquinolines [75]. Very recently, Zhu and coworkers used an analogous gold(III) complex to catalyze the reactions similar to those reported by the Rueping group (Scheme 9) [76]. A 5 W blue LED was used as the light source. One advantage of using the gold complex over [Ru(bpy)_3_](PF_6_)_2_ is that long-chain aliphatic ketones work much better using the former catalyst. Other types of pronucleophiles such as malonates are also effective in the Mannich reaction.

**Scheme 10:**
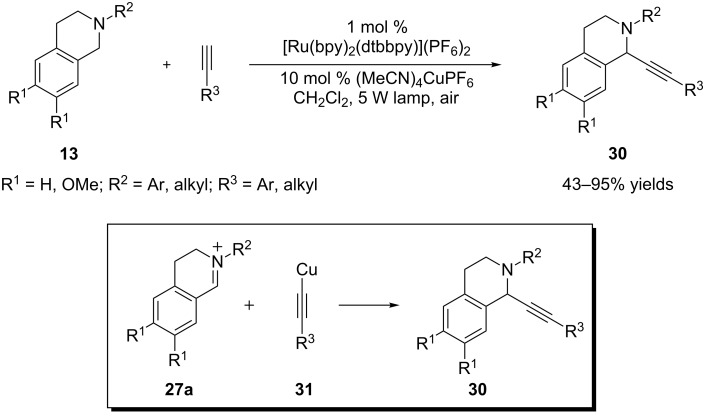
Merging Ru-based photoredox catalysis and Cu-catalyzed alkynylation reaction.

The Rueping group extended the concept of dual catalysis by merging visible light photocatalysis with a metal-catalyzed process (Scheme 10) [77]. To make this approach work, several hurdles need to be addressed. First, a labile carbon–metal bond is desired in order to have an efficient turnover of the metal. Second, the metal complex needs to be compatible with the strongly reducing intermediates (e.g., superoxide) produced in the photocatalytic cycle. Third, the rates of the two catalytic cycles have to be comparable, as iminium ions are known to be converted to amides by superoxide [65–66]. Rueping and coworkers discovered that using a weak light source (5 W fluorescent bulb), copper acetylide **31**, formed in situ by (MeCN)_4_CuPF_6_, was added efficiently to the photogenically-produced iminium ion **27a**, thus achieving the formation of Csp^3^–Csp bonds.

**Scheme 11:**
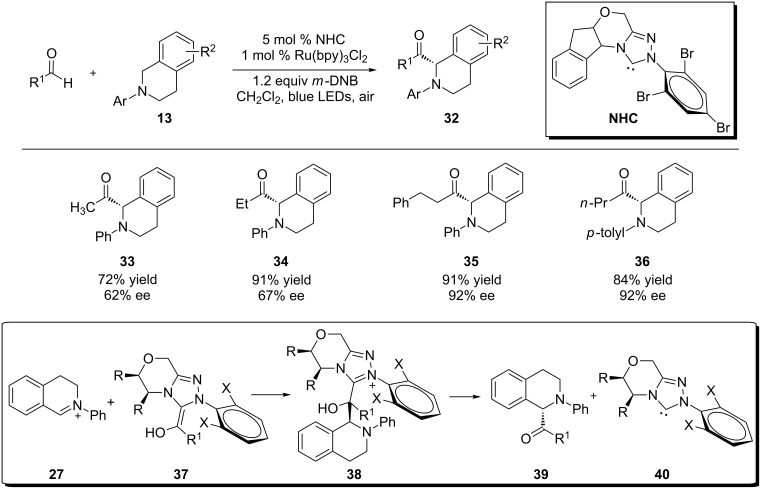
Merging Ru-based photoredox catalysis and NHC catalysis.

Rovis and coworkers recently developed another mode of dual catalysis involving visible light photocatalysis with chiral *N*-heterocyclic carbene catalysis, which allows catalytic asymmetric α-acylation of *N*-aryltetrahydroisoquinoline **13** with an aliphatic aldehyde (Scheme 11) [78]. In the presence of a *N*-heterocyclic carbene (NHC), the aldehyde is converted to a chiral acyl anion or homoenolate equivalent **37**, which is then added to the iminium ion **27** to form Csp^3^–Csp^2^ bonds asymmetrically. It is interesting to note that the use of *m*-dinitrobenzene (*m*-DNB) is critical to achieve the desirable conversion and yield of the expected product **32**. *m*-DNB is proposed to act as an electron acceptor to promote an oxidative quenching cycle of Ru(bpy)_3_^2+^* to Ru(bpy)_3_^3+^. *N*-aryltetrahydroisoquinoline **13** is then oxidized by Ru(bpy)_3_^3+^_._ This is in contrast to the majority of reported examples in which the conversion to the iminum ion such as **27** is realized in a reductive quenching cycle of Ru(bpy)_3_^2+^* to Ru(bpy)_3_^1+^, where *N*-aryltetrahydroisoquinoline **13** is oxidized by Ru(bpy)_3_^2+^* instead.

**Scheme 12:**
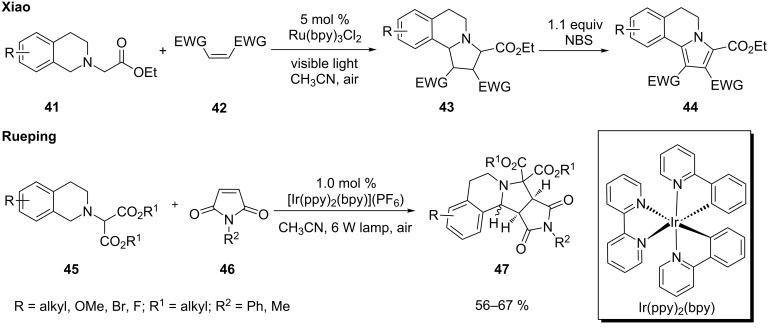
1,3-Dipolar cycloaddition of photogenically formed azomethine ylides.

Xiao [79] and Rueping [80] independently reported that when tetrahydroisoquinolines (e.g., **41** and **45**) were substituted with a methylene group attached to one or two esters, the initially formed iminium ions were readily converted to azomethine ylides. They subsequently underwent 1,3-dipolar cycloaddition with a range of dipolarophiles to form fused pyrrolidines **43** and **47** (Scheme 12). Xiao also showed that the pyrrolidine ring of **43** could be further oxidized to a fused pyrrole **44** under the same photoredox conditions or by treatment with NBS. Both Ru(bpy)_3_Cl_2_ and Ir(bpy)(ppy)_2_ were found to be effective catalysts.

**Scheme 13:**
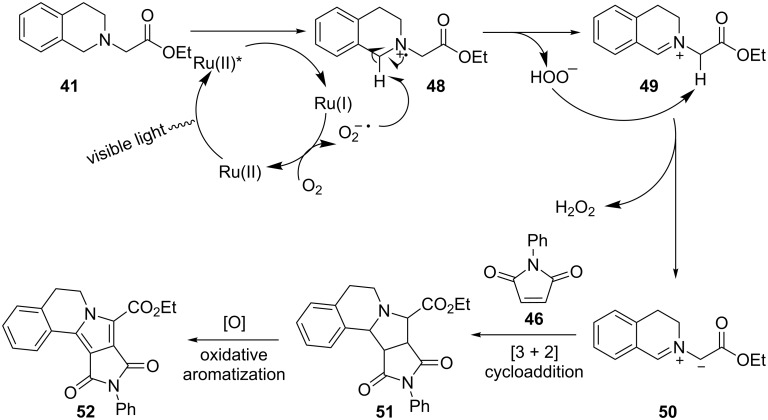
Plausible mechanism for photoredox 1,3-dipolar cycloaddition.

A plausible mechanism for the 1,3-dipolar cycloaddition is shown in Scheme 13. The reaction commences with oxidation of tetrahydroisoquinoline **41** to amine radical cation **48** by the photoexcited state of Ru^2+^. Subsequently, abstraction of a hydrogen atom α to the nitrogen atom of **48** yields iminium ion **49**, which is then converted to azomethine ylide **50** by loss of a proton. 1,3-Dipolar cycloaddition of **50** with a dipolarophile **46** furnishes fused pyrrolidine **51** that is further oxidized to pyrrole **52**.

The Zhu group discovered that the use of α-ketoester **53** as a pronucleophile to intercept the iminium ion of **13** triggered a new cascade reaction en route to fused isoxazolidine **54** in excellent diastereoselectivity (>20:1, Scheme 14) [81]. Alcohols were found to be the solvent of choice for this reaction. Among the three alcohols screened, iPrOH was more effective than MeOH or EtOH, resulting in a shorter reaction time. The addition of a catalytic amount of TfOH had marginally beneficial effects on the reaction time and yields. Interestingly, depending on the electronic character of the *N*-aryl group, [Ir(ppy)_2_(dtbbpy)](BF_4_) or Ru(bpy)_3_Cl_2_ was used to obtain optimal yields. The former catalyst worked better with electron-poor *N*-aryl groups while the latter was more effective for electron-rich *N*-aryl groups.

**Scheme 14:**
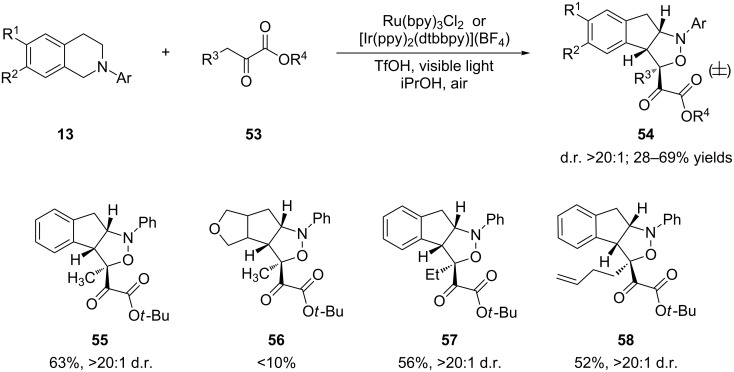
Photoredox-catalyzed cascade reaction for the synthesis of fused isoxazolidines.

**Scheme 15:**
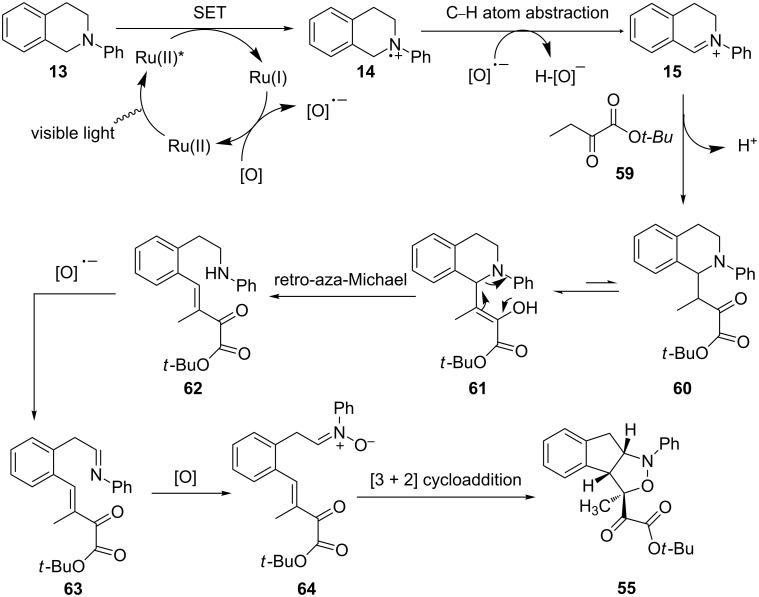
Plausible mechanism for the photoredox-catalyzed cascade reaction.

The authors proposed a possible mechanism that starts with reductive quenching of the photoexcited state of Ru(II) or Ir(III) by *N*-phenyltetrahydroisoquinoline **13** (Scheme 15). The initially formed amine radical cation **14** is converted to iminium ion **15** by abstraction of a hydrogen atom directly. The addition of the enol form of α-ketoester **59** to **15** furnishes the Mannich adduct **60**. A retro-aza-Michael reaction via enol **61** allows cleavage of the C–N bond to yield secondary aniline **62**. Aniline **62** is first oxidized to imine **63**, which is further oxidized to nitrone **64**. Finally, an intramolecular 1,3-dipolar cycloaddition of **64** furnishes isoxazolidine **55**.

**Scheme 16:**
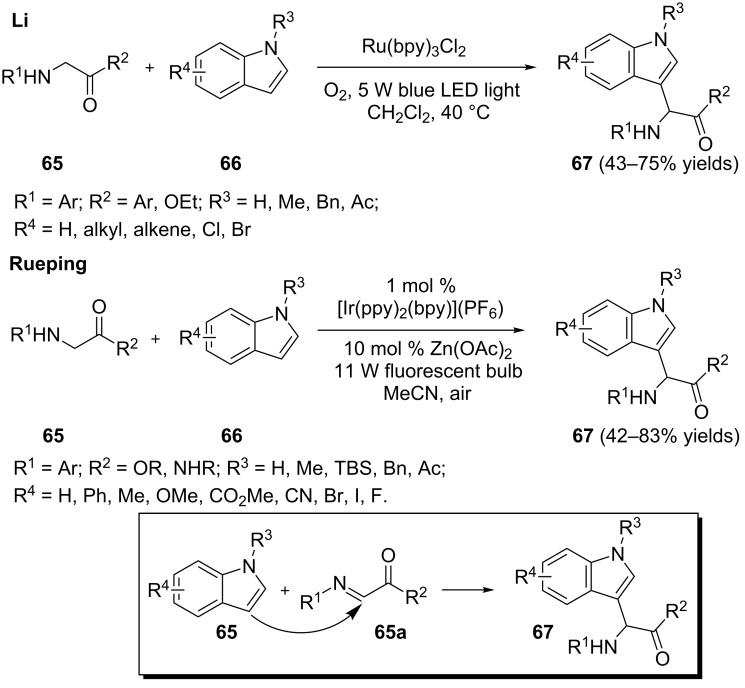
Photoredox-catalyzed α-arylation of glycine derivatives.

Tetrahydroisoquinolines are arguably the most exploited amines in visible light photoredox catalysis. However, efforts towards expanding the scope of amines have been recently reported. Li [82] and Rueping [83] independently reported that *N*-arylglycine derivatives **65** are viable substrates (Scheme 16). They are presumably converted to imines **65a** that are intercepted by indoles to give the Mannich-like adducts **67**. The conditions used by Li were 10 mol % Ru(bpy)_3_Cl_2_ and 1 atm O_2_ at 40 °C with a 5 W blue LED as the light source. *N*-arylglycine derivatives **65**, including esters and ketones, were successfully converted to the products **67**. Rueping used Ir(ppy)_2_(bpy)PF_6_ as the photocatalyst, air, and an 11 W fluorescent bulb as the light source. Additionally, Zn(OAc)_2_ was employed as a Lewis acid cocatalyst. It was postulated that Zn(OAc)_2_ facilitates the conversion of the initially formed amine radical cation to the imine **65a** and subsequently activates the imine for nucelophilic attack. *N*-arylglycine esters and *N*-arylglycine derived dipeptides worked quite well under these conditions. However, the ketones failed to provide the desired products.

**Scheme 17:**
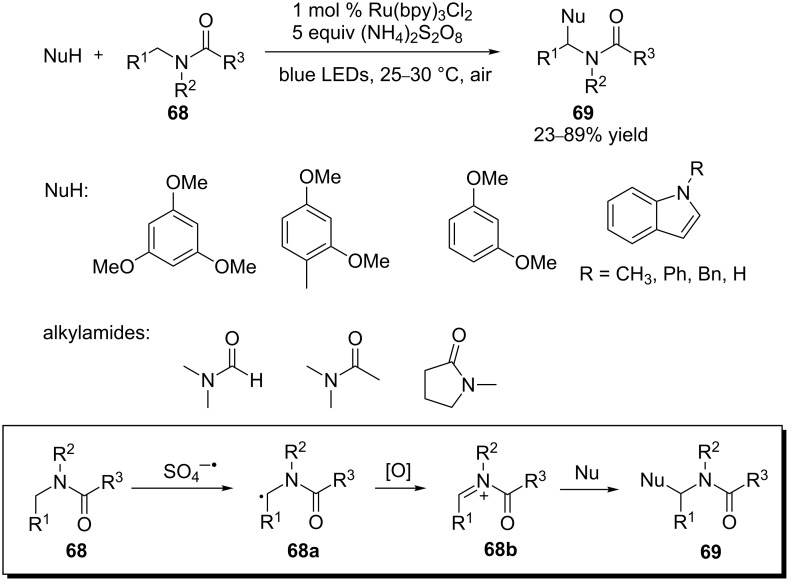
Photoredox-catalyzed α-arylation of amides.

Amides **68** are generally much more difficult to be oxidized than amines. Their reduction potentials range from 1.2–1.5 V (vs SCE) for tertiary amides to 2.0 V (vs SCE) for primary amides [84] which makes them less susceptible to oxidation by the photoexcited state of Ru(II) or Ir(III) complexes (Ru(bpy)_3_^2+^*/Ru^1+^: 0.77 V vs SCE; Ir(ppy)_2_(dtbbpy)^+^*/Ir(ppy)_2_(dtbbpy), 0.66 V vs SCE) [35]. Stephenson and coworkers devised a strategy by reversing the order of oxidation and C–H abstraction to overcome this issue (Scheme 17) [85]. The first intermediate formed is a strongly reducing α-amino radical **68a** that is oxidizable by the photoexcited state of Ru(II) or Ir(III). The α-amino radical **68a** is formed via C–H abstraction by the sulfate radical anion (SO_4_^−·^), which is generated by exposure of Ru^2+^* to persulfate (S_2_O_8_^2−^), an oxidative quencher. Electron-rich arenes and indoles are then added to the *N*-acyliminium ions **68b** to provide the amidoalkylation products **69**. Alternatively, the use of only persulfate at 55 °C afforded the same products. However, higher yields and better selectivities were generally observed with the photocatalytic process.

#### Intercepted by nitrogen, oxygen, or phosphorus nucleophiles

**Scheme 18:**
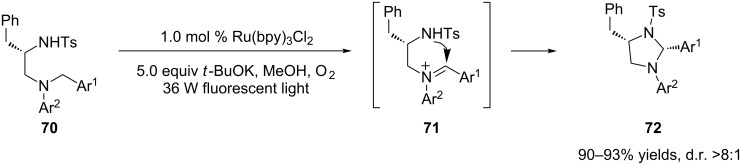
Intramolecular interception of iminium ions by sulfonamides.

In addition to carbon nucleophiles, heteronucleophiles including nitrogen, oxygen, and phosphorus are susceptible to interception of the photogenically formed iminium ions. The Xiao group developed a highly diastereroselective route to substituted tetrahydroimidazoles **72** based on intramolecular interception of the iminium ions by a tethered sulfonamide (**71**, Scheme 18) [86]. Ru(bpy)_3_Cl_2_ was employed as the photocatalyst with oxygen as the stoichiometric oxidant. The use of a base in an alcohol solvent, such as MeOH, was also the key to the success of this reaction. The diastereoselectivities were greatly improved by prolonging the reaction time, which would allow for epimerization leading to the thermodynamically more stable products. The starting materials, 1,2-diamines **70**, were readily prepared from natural amino acids in enantiomerically pure form.

**Scheme 19:**
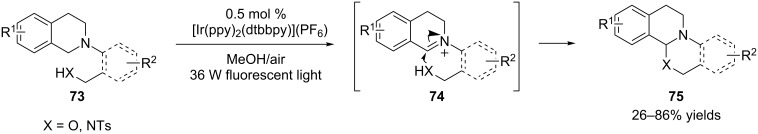
Intramolecular interception of iminium ions by alcohols and sulfonamides.

Xiao and coworkers then applied the same strategy to prepare two other types of heterocycles, isoquino[2,1-*a*][3,1]oxazine and isoquino[2,1-*a*]pyrimidine (**75**, Scheme 19) [87]. The use of [Ir(ppy)_2_(dtbbpy)](PF_6_) with air as the external oxidant was found to be optimal for catalyzing the reaction. Later, the Marvin group reported an identical synthesis of isoquino[2,1-*a*][3,1]oxazine using Ru(bpy)_3_Cl_2_ instead [88]. The tethered nucleophiles, primary alcohols or sulfonamides, are part of the *N*-aryl group of tetrahydroisoquinolines **73**. Similar to the synthesis of tetrahydroimidazoles **72**, MeOH was the optimal solvent. No external base was needed when the alcohol was the nucleophile. However, if the sulfonamide was the nucleophile, an external base such as *t*-BuOK was required presumably to increase the nucleophilicity of the sulfonamide.

**Scheme 20:**
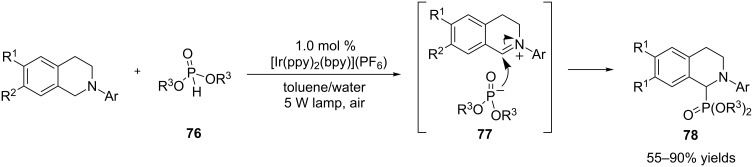
Intermolecular interception of iminium ions by phosphites.

The Rueping group trapped the iminium ions using phosphites **76** to produce α*-*amino phosphonates **78** (Scheme 20) [89]. [Ir(ppy)_2_(bpy)](PF_6_) was found to be the most effective catalyst. Interestingly, a biphasic mixture of toluene and water turned out to be the optimal solvent. The often-observed byproducts, amides derived from over-oxidation of the iminium ions, were suppressed [65–66]. The reactions were also sensitive to the steric and electronic nature of phosphites. Phosphites are quite acidic; their p*K*_a_s are similar to alcohols. Less sterically hindered phosphites reacted faster as did more acidic phosphites (e.g., diphenylphosphite).

**Scheme 21:**
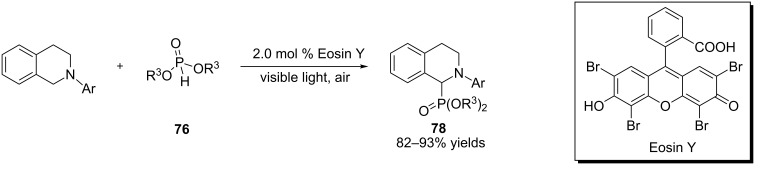
Photoredox-catalyzed oxidative phosphonylation by Eosin Y.

The König group applied the organic dye Eosin Y as the photocatalyst to catalyze the same reactions (Scheme 21) [67]. The reactions were irradiated in DMF with green LED light, which overlapped with the λ_max_ of Eosin Y. The yields are comparable for the two catalyst systems, but the reactions catalyzed by Eosin Y are much faster (note: the conclusion is based on 2 mol % Eosin Y vs 1 mol % [Ir(ppy)_2_(bpy)](PF_6_)).

### α-Amino radicals

α-Amino radicals are another class of downstream intermediates produced from amine radical cations. They are also the key intermediate in one of two potential pathways for the conversion of amine radical cations to iminium ions (Scheme 1). In contrast to the electrophilic nature of iminum ions, α-amino radicals are nulceophilic. They tend to add to Michael acceptors in a 1,4 fashion. Since the addition is overall redox neutral, no external oxidant is required. Most of these addition reactions are conducted under degassing conditions. This is in contrast to the chemistries involving iminium ions, which are often performed with exposure to air or oxygen. The reactivity umpolung at the carbon α to the nitrogen atom has expanded the repertoire of amine radical cations’ modes of reactivity. Compared to iminium ions, α-amino radicals have been much less exploited as synthetic intermediates. Their synthetic applications remain limited.

**Scheme 22:**
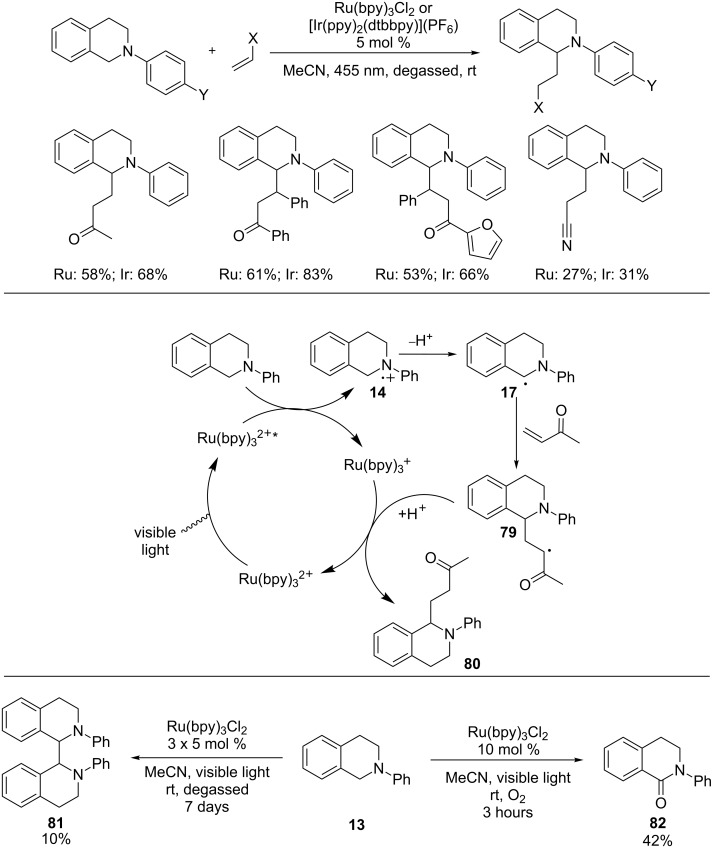
Conjugated addition of α-amino radicals to Michael acceptors.

Pandey and Reiser revealed that α-amino radicals derived from *N*-aryltetrahydroisoquinolines were added intermolecularly to Michael acceptors (Scheme 22) [66]. A blue LED was used as the light source. Both Ru(bpy)_3_Cl_2_ and [Ir(ppy)_2_(dtbbpy)](PF_6_) were found to catalyze the reactions. However, in some of the examples, the Ir catalyst gave better yields. Mechanistically, reductive quenching of the photoexcited Ru(II) or Ir(III) complex by *N*-aryltetrahydroisoquinolineamine yields amine radical cation **14**, which is converted to α-amino radical **17**. Conjugated addition of **17** to methyl vinyl ketone produces radical **79**, which is reduced by the Ru(I) or Ir(II) complex with concomitant regeneration of the Ru(II) or Ir(III) complex. Protonation of the resulting enolate furnishes the adduct **80**, thus completing the catalytic cycle. The authors performed two control studies to probe the involvement of the α-amino radical **17**. The first study was to irradiate *N*-phenyltetrahydroisoquinoline **13** in the absence of the Michael acceptor under otherwise identical conditions. The dimer, **81**, was formed as a mixture of diastereomers. The second study involved irradiation of *N*-phenyltetrahydroisoquinoline **13** only without degassing the reaction solution. The amide, **82**, was produced instead. Both findings lend credence to the intermediacy of the α-amino radical **17**.

**Scheme 23:**
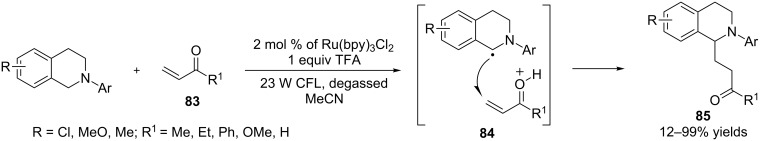
Conjugated addition of α-amino radicals to Michael acceptors assisted by a Brønsted acid.

The Yoon group independently discovered that the efficiency of the same Michael reaction was greatly improved in the presence of a Brønsted acid (Scheme 23) [90]. Some of the improvements included shorter reaction time, higher yields, and use of a weaker light source (CFL). The most effective acid catalysts, of which TFA was found to be optimal, lie within a narrow range of p*K*_a_ values. The authors suggested that TFA protonates the enone **83**, thus accelerating the addition of the α-amino radical to the enone (**84**).

**Scheme 24:**
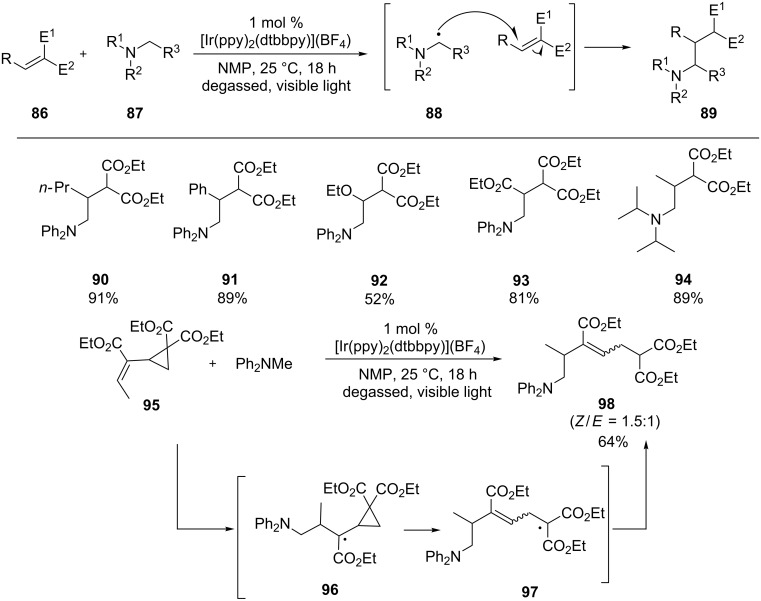
Conjugated addition of α-amino radicals derived from anilines to Michael acceptors.

The Nishibayashi group reported that α-amino radicals generated from a different class of amines, anilines **87**, were also added intermolecularly to Michael acceptors **86** (Scheme 24) [91]. In this reaction, the Michael acceptors **86** were limited to those activated by two electron-withdrawing groups. [Ir(ppy)_2_(dtbbpy)](BF_4_) was found to be the most effective photocatalyst. Solvents were also critical to the outcome of the reaction; NMP produced much higher yields of the products **89** than DMF, while no products were formed in MeCN or MeOH. The authors interrogated the intermediacy of the α-amino radical **88** by treatment of diphenylmethylaniline with a Michael acceptor incorporating a cyclopropyl ring **95**. The ring-opening product **98** was isolated in 64% yield, which is consistent with the involvement of the α-amino radical **88**.

**Scheme 25:**
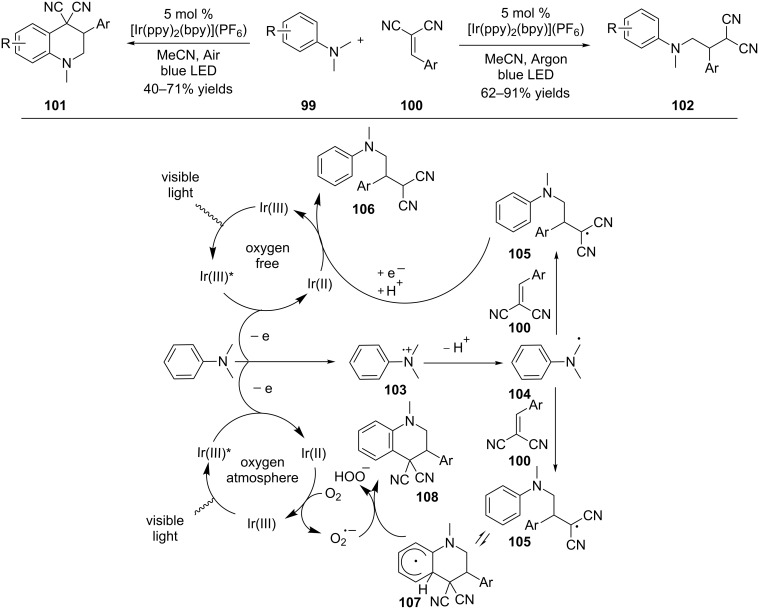
Oxygen switch between two pathways involving α-amino radicals.

Oxygen has been suggested to play multiple roles in the oxidation of amines under photoredox conditions (vide supra). The Rueping group recently reported a new role that oxygen played in the intermolecular addition of α-amino radicals to Michael acceptors (Scheme 25) [92]. Oxygen was found to act as a chemical switch to two competing reaction pathways from the same starting anilines. Irradiation of a degassed solution of aniline **99**, 2-benzylidenemalononitrile **100**, 5 mol % Ir(ppy)_2_(bpy)](PF_6_) in MeCN furnished the typical Michael adduct **102**. This result is similar to those reported by Pandey and Reiser [66], and Nishibayashi [91]. However, when the irradiation was conducted in the presence of air, a different reaction pathway occurred, resulting in the formation of *N*-alkyltetrahydroquinoline **101**. The two reaction pathways diverge from the radical intermediate **105** generated from the Michael addition of α-amino radical **104** to 2-benzylidenemalononitrile **100**. Without oxygen, the radical undergoes a one-electron reduction by Ir(II) to produce a stabilized anion, which is protonated to afford the Michael adduct **106**. Alternatively, the radical is added onto arene to form a cyclohexadienyl radical **107**. This step is reversible in the absence of oxygen. However, in the presence of oxygen, superoxide is formed via one-electron reduction of oxygen by Ir(II). The cyclohexadienyl radical **107** is converted to the cyclization product **108** irreversibly by giving one electron and one proton to the superoxide.

**Scheme 26:**
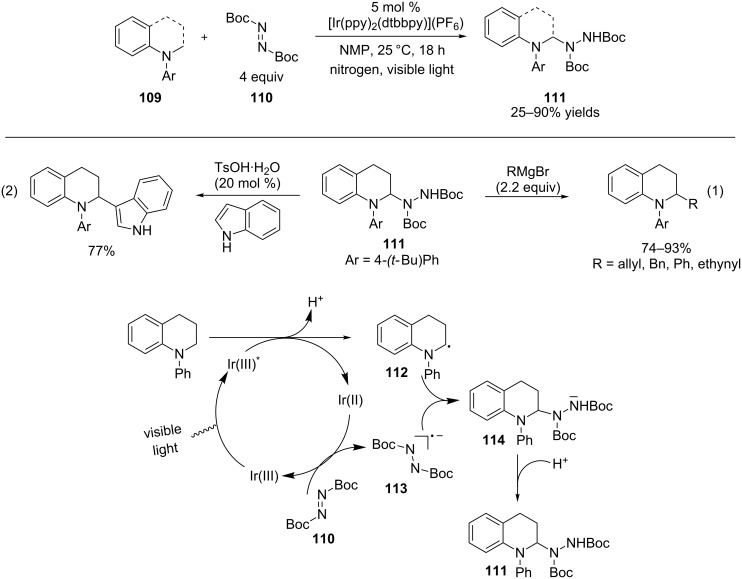
Interception of α-amino radicals by azodicarboxylates.

The Nishibayashi group also successfully trapped α-amino radicals derived from *N*-aryltetrahydroquinolines and *N*-arylindolines using di-*tert*-butyl azodicarboxylate **110** to form *N*,*N*-acetal products **111** (Scheme 26) [93]. Functionalization of the sp^3^ C–H bond α to the nitrogen atom in tetrahydroquinolines and indolines via iminium ions is challenging because the corresponding iminium ions are enolizable and thus tend to tautomerize to enamines [94–95] and/or aromatize [96–97]. The authors adopted a strategy to bypass the iminium ions and use α-amino radicals such as **112** instead to construct C–N bonds. Treatment of *N*,*N*-acetal product **111** with Grignard reagents (Scheme 26, entry 1) or indoles in the presence of TsOH (Scheme 26, entry 2) provided nucleophilic substitution products at the α carbon. This provides an indirect approach for α-C–H functionalization of *N*-aryltetrahydroquinolines and *N*-arylindolines. Based on the feasibility of oxidation of aromatic amines as well as reduction of di-*tert*-butyl azodicarboxylate (**110**) by the photoexcited Ir(III) complex [98–99], the authors favored a mechanism that does not involve the direct addition of α-amino radical **112** to di-*tert*-butyl azodicarboxylate (**110**). Oxidation of *N*-phenyltetrahydroquinoline by the photoexcited Ir(III) complex followed by deprotonation provides α-amino radical **112** with the concomitant formation of the Ir(II) complex. Di-*tert*-butyl azodicarboxylate (**110**) is reduced by the Ir(II) complex to generate radical anion **113**, which couples with α-amino radical **112** to yield nitrogen anion **114**. Concurrently, the Ir(III) complex is regenerated. Protonation of **114** furnishes *N*,*N*-acetal **111**.

**Scheme 27:**
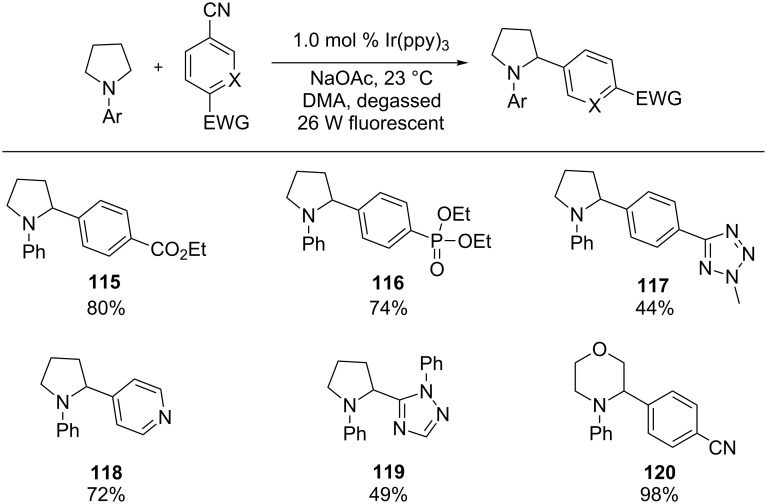
α-Arylation of amines.

α-Amino radicals have been mainly used in conjugate addition reactions. Recently, the MacMillan group has nicely expanded the scope of the reactions to include addition of the radicals to aryl rings (Scheme 27) [100]. Using Ir(ppy)_3_ as the photocatalyst and a 26 W fluorescent light bulb as the light source, cyclic amines with a variety of ring sizes and acyclic amines underwent the α-arylation reaction to provide benzylic amines. The arylating reagents were benzonitriles substituted with an electron-withdrawing group. The nitrile group functioned as the leaving group. In some classes of five-membered heteroaromatics, a chloride was capable of replacing the nitrile group as the leaving group.

**Scheme 28:**
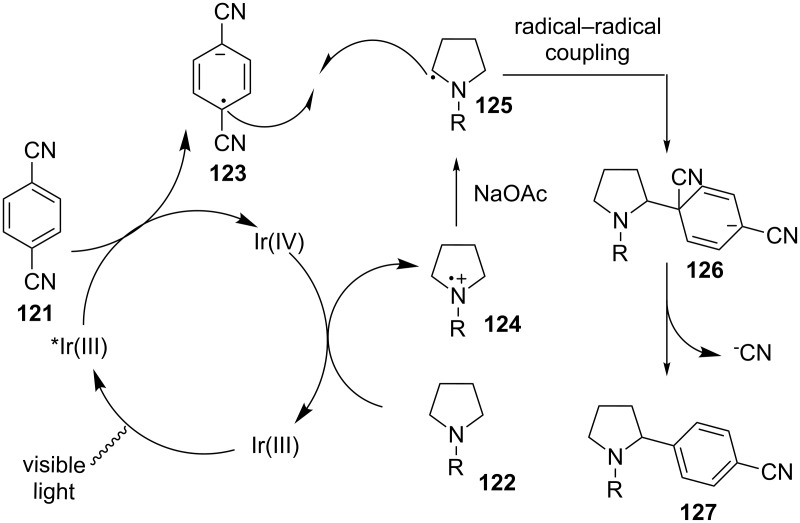
Plausible mechanism for α-arylation of amines.

The authors proposed a mechanistic pathway that is initiated by oxidative quenching of the photoexcited state of Ir(ppy)_3_ by benzonitrile **121** to generate radical anion **123** and Ir^4+^(ppy)_3_ (Scheme 28). Amine **122** is then oxidized to amine radical cation **124** by Ir^4+^(ppy)_3_ that is reduced to the initial catalyst, Ir(ppy)_3_. Deprotonation of amine radical cation **124** by NaOAc produces α-amino radical **125**, which is coupled with radical anion **123** to form the key C–C bond in **126**. Finally, aromatization via expulsion of the nitrile group provides benzylic amine **127**.

### Cleavage of C–C and N–N bonds

**Scheme 29:**
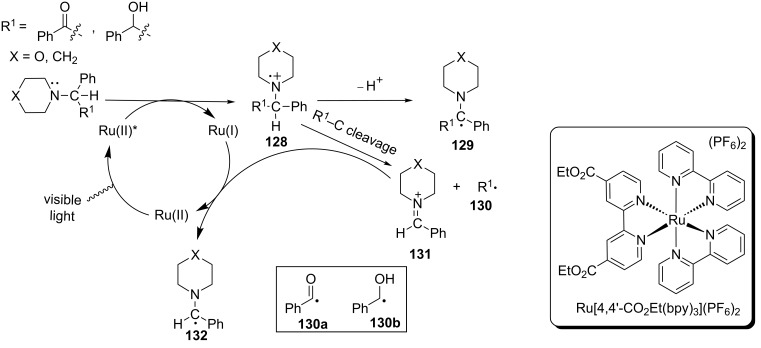
Photoinduced C–C bond cleavage of tertiary amines.

The dominant reaction pathway involving the photogenically formed amine radical cations is deprotonation at the carbon α to the nitrogen atom to produce the strongly reducing α-amino radicals (e.g., **128** to **129**). α-Amino radicals can be then intercepted by Michael acceptors or undergo one-electron oxidation to yield iminium ions (vide supra). An alternative yet much less exploited reaction pathway concerning amine radical cations **129** is the cleavage of the C–C bond α to the nitrogen atom to generate a neutral carbon radical (e.g., **130**) and an iminium ion (e.g., **131**). The iminum ion is subsequently reduced to α-amino radical **132** by Ru(I). Back in 1986, the Whitten group established this pathway by irradiation of three substituted tertiary amines with Ru[4,4’-CO_2_Et(bpy)]_3_(PF_6_)_2_ respectively using visible light (Scheme 29) [101]. The identity of carbon radicals **130a** and **130b** was established by trapping them with a spin trap and then analyzing using EPR. Additionally, detection of benzaldehyde by HPLC and VPC provided further evidence for their formation. In contrast, no products from the amine half (e.g., **131** and **132**) were detected.

**Scheme 30:**
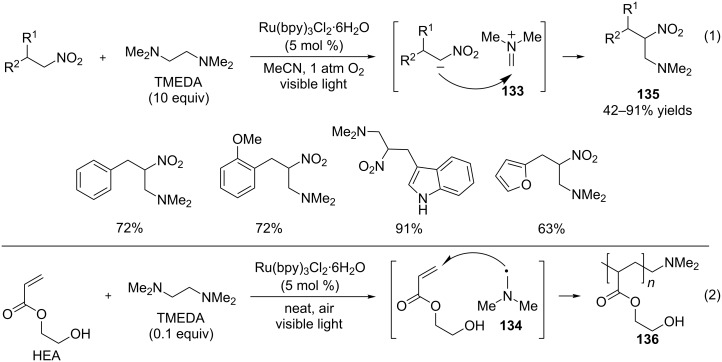
Photoredox cleavage of C–C bonds of 1,2-diamines.

Li and Wang recently applied this cleavage reaction to 1,2-diamines, simultaneously generating two classes of synthetically useful intermediates, iminium ions (e.g., **133**, Scheme 30) and α-amino radicals (e.g., **134**, Scheme 30) [102]. The authors then exploited the synthetic utility of these two classes of intermediates. Irradiation of nitroalkanes, TMEDA, and Ru(bpy)_3_Cl_2_ in 1 atm oxygen afforded the aza-Henry products **135**, presumably by trapping the Me_2_N=CH_2_ iminium ion **133** that is formed by cleaving TMEDA (Scheme 30, entry 1). Separately, irradiation of 2-hydroxyethylacrylate (HEA), TMEDA, and Ru(bpy)_3_Cl_2_ in air produced a polymer incorporating a dimethylamino group (**136**, Scheme 30, entry 2). The dimethylamino radical, the other intermediate generated by cleaving TMEDA, most likely induced the polymerization. The chemistries involving the iminium ions and α-amino radicals, generated under visible light photoredox conditions, are often limited by the substrate scope of the amine precursors, since aromatic amines are typically required (vide supra). The cleavage reaction, as demonstrated by Li and Wang’s work, has the potential to produce different types of iminium ions and α-amino radicals that are not accessible by oxidizing amines directly.

**Scheme 31:**
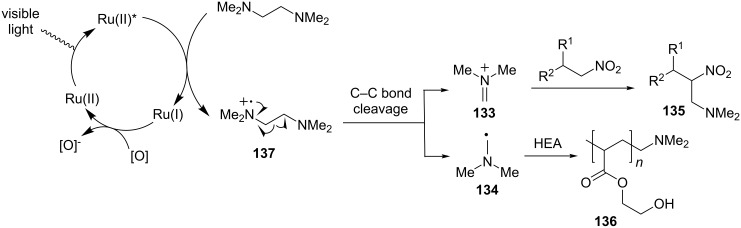
Proposed mechanism photoredox cleavage of C–C bonds.

The reaction is proposed to proceed through the initial oxidation of TMEDA to amine radical cation **137** by the photoexcited state of the Ru(II) complex (Scheme 31). Amine radical cation **137** subsequently induces cleavage of the C–C bond α to the nitrogen atom to form iminium ion **133** and α-amino radical **134** concurrently. By carefully selecting reagents/conditions, either reactive intermediate can selectively participate in the designated reaction. As shown in Li and Wang’s work, iminium ion **133** is intercepted by nitroalkane to afford the aza-Henry product **135** while α-amino radical **134** is used to initialize radical polymerization of HEA.

**Scheme 32:**
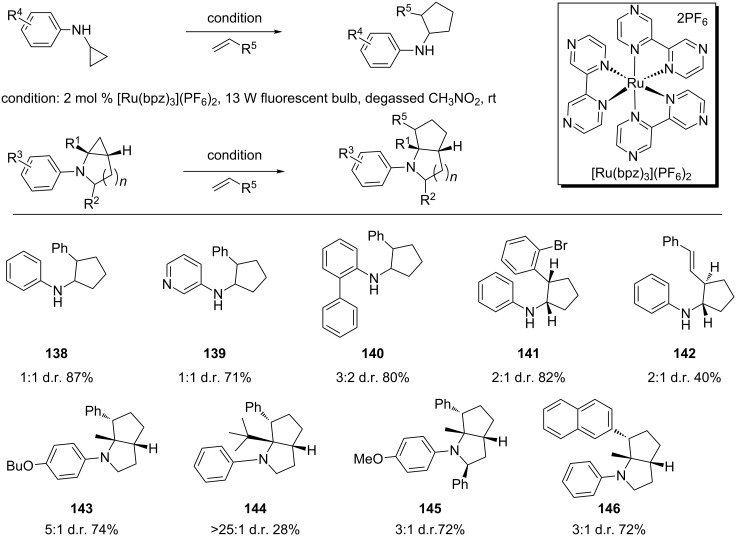
Intermolecular [3 + 2] annulation of cyclopropylamines with olefins.

Because of ring strain, cyclopropanes are prone to ring opening via cleavage of one of the three C–C bonds. The resulting reactive intermediates have been shown to participate in a number of synthetic/mechanistic applications [103–104]. One of these applications is a radical clock, which is centered on the cyclopropylcarbonyl to homoallyl radical rearrangement [105]. A homologous rearrangement based on the amine radical cation of *N*-cyclopropylanilines permits cleavage of the C–C bond α to the nitrogen atom but generating a γ-carbon radical iminium ion (distonic ion) [106]. We have applied Ru(bpz)_3_(PF_6_)_2_-catalyzed photooxidation of *N*-cyclopropylanilines to induce this rearrangement reaction. The resulting distonic ion was then intercepted by alkenes to produce [3 + 2] annulation products (Scheme 32) [107]. An aryl group on the amine was required for the reaction. Both mono- and bicyclic cyclopropylanilines were viable substrates to provide the annulation products in good to excellent yields. The former gave little to poor diastereoselectivity whereas the later produced modest diastereoselectivity. The reaction has 100% atom economy. It is also overall redox-neutral and thus does not require an external oxidant.

**Scheme 33:**
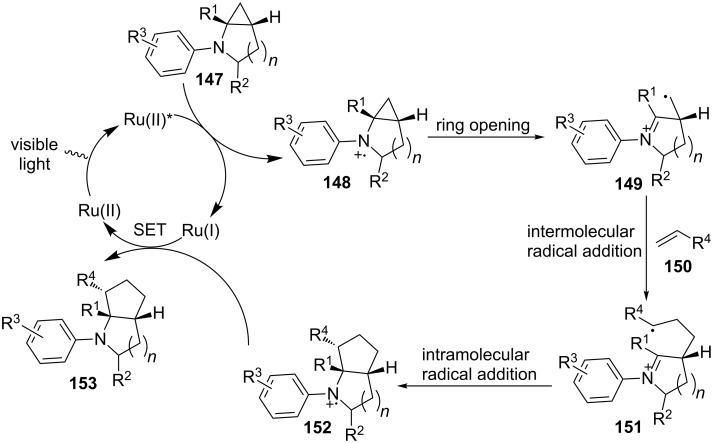
Proposed mechanism for intermolecular [3 + 2] annulation.

We believe that the annulation reaction proceeds first via reductive quenching of the photoexcited state of Ru(II) by cyclopropylaniline **147** to generate amine radical cation **148** and Ru(I) (Scheme 33). Amine radical cation **148** then triggers the ring opening to release the ring strain while producing a distonic ion **149** with a primary radical. Distonic ion **149** is added via a Giese-type radical addition to an alkene, yielding a more stable distonic ion **151** with a secondary radical. Intramolecular addition of the secondary radical to the iminium ion furnishes a new amine radical cation **152**. Finally, amine radical cation **152** is reduced by Ru(I) to provide the annulation product **153** and regenerate Ru(II), thus completing the catalytic cycle.

**Scheme 34:**
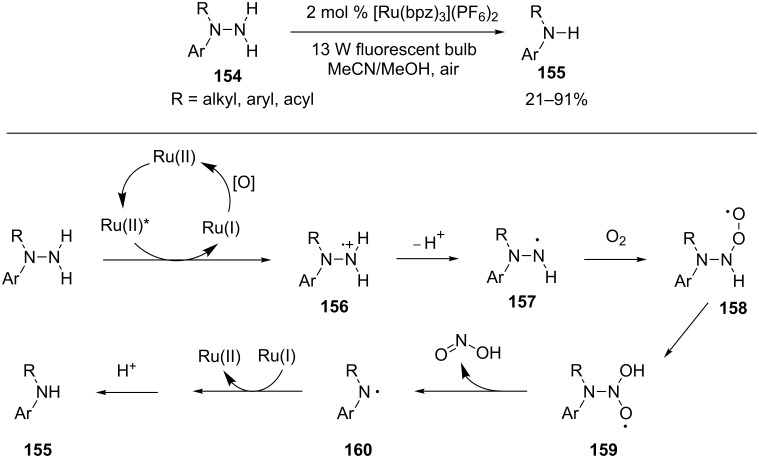
Photoinduced clevage of N–N bonds of aromatic hydrazines and hydrazides.

Our group also realized cleavage of N–N bonds by irradiation of aromatic hydrazines or hydrazides in the presence of Ru(bpz)_3_(PF_6_)_2_ and air (Scheme 34) [108]. A 13 W compact fluorescent light was sufficient as the light source. *N*,*N*-disubstituted hydrazines and hydrazides were suitable substrates provided that at least one of the two substituents on the nitrogen atom was an aryl group. Electron-richer hydrazines were found to be more reactive than hydrazides. This is consistent with our expectation that, similar to amines, hydrazines and hydrazides act as an electron donor to reductively quench the photoexcited Ru(II) complex. The photoexcited state of Ru(bpz)_3_ (E_1/2_*^II/I^ = 1.45 V vs SCE) is more oxidizing than that of Ru(bpy)_3_ (E_1/2_*^II/I^ = 0.77 V vs SCE). However, the two catalysts showed a divergent pattern of reactivity in the reaction. Ru(bpy)_3_ was the more active catalyst for hydrazines, whereas Ru(bpz)_3_ was more active for hydrazides. The use of MeOH in addition to CH_3_CN significantly shortened the reaction time for less reactive hydrazides, but showed little effect for hydrazines. We believe that the cleavage reaction is initialized via the oxidation of hydrazines or hydrazides to a amine radical cation **156** by the photoexcited Ru(II) complex. Deprotonation of the amine radical cation **156** produces a neutral nitrogen radical **157** that reacts with oxygen to furnish the radical **158**. The radical **158** then rearranges to a new oxygen-based radical **159**, which undergoes a cleavage reaction to yield nitrous acid and a secondary amine radical **160**. Finally, one-electron reduction of the amine radical by Ru(I), followed by protonation provides a secondary amine **155**.

## Conclusion

Visible light photoredox catalysis provides a unique way to activate small molecules such as amines. The dual nature of the photocatalyst’s photoexcited state as both oxidant and reductant allows accepting or donating one electron strictly dependent upon the small molecules encountered. Amines typically act as an electron donor to reductively quench the photoexcited state while they are oxidized to the corresponding amine radical cations. The resulting nitrogen radical cations are highly useful reactive intermediates that are capable of initializing multiple downstream pathways leading to diverse synthetic intermediates such as electrophilic iminium ions, nucleophilic α-amino radicals, and distonic ions possessing both an iminium ion and a carbon radical. Interception of these intermediates allows a variety of synthetic transformations to produce a diverse array of amines. Moreover, visible light photoredox catalysis has been merged with other types of catalysis, including enamine catalysis, *N*-heterocyclic carbene (NHC) catalysis, or copper acetylide formation. This dual catalysis approach has significantly expanded the type of bonds that can be formed, particularly bonds formed asymmetrically. In summary, the utility of amine radical cations formed via photooxidation of the amines has been amply demonstrated in a number of synthetic methods. With the organic community’s increasing interest in visible light photoredox catalysis, new and innovative applications of this reactive intermediate will continue to develop.
